# Enhanced pre-pubertal nutrition upregulates mitochondrial function in testes and sperm of post-pubertal Holstein bulls

**DOI:** 10.1038/s41598-020-59067-3

**Published:** 2020-02-10

**Authors:** Chinju Johnson, Alysha Dance, Igor Kovalchuk, John Kastelic, Jacob Thundathil

**Affiliations:** 1 Department of Production Animal Health, Faculty of Veterinary Medicine, University of Calgary, Calgary, AB T2N 4N1 Canada; 2 Department of Biological Sciences, University of Lethbridge, Lethbridge, AB TIK 3M4 Canada

**Keywords:** Developmental biology, Molecular biology

## Abstract

Supplemental energy and protein during calf-hood (2–30 wk) in dairy bulls hastened puberty (~1 mo), upregulated steroid biosynthesis, concentrations of reproductive hormones and Sertoli cell maturation, with larger testes and greater sperm production (~25%) in mature bulls. The objective was to evaluate effects of feeding high (20.0% crude protein [CP], 67.9% total digestible nutrients [TDN]), control/medium (17.0% CP, 66.0% TDN) and low (12.2% CP, 62.9% TDN) diets from 2 to 30 wk on post-pubertal testes of Holstein bulls. Based on RNA sequencing, 497 and 2961 genes were differentially expressed (P < 0.1) in high- vs low- and high- vs medium-diet groups, respectively. According to KEGG analysis, oxidative phosphorylation and ribosome pathways were upregulated in high- vs medium- and low-diet groups, with majority of upregulated genes encoding for essential subunits of complex I, III, IV and V of OXYPHOS pathway. In addition, mitochondrial translation, mitotic nuclear division and cell division were enriched in high- vs medium-diet groups. Consistent with these results, a greater percentage of sperm from high-diet bulls were progressively motile and had normal mitochondrial function compared to medium-diet sperm (P < 0.1). Thus, enhanced early life nutrition upregulated mitochondrial function in testes and sperm of post-pubertal Holstein bulls.

## Introduction

Enhanced early-life nutrition hastens puberty, with ~25% increases in testis size and sperm production in dairy and beef bulls^1,2^. The underlying endocrine basis is profound increases in luteinizing hormone (LH) pulse frequency and insulin like growth factor (IGF-I) concentrations in blood^3,4^. In a cohort-based study in Sweden, low nutrition during the pre-pubertal period in men reduced susceptibility to heart disease in their offspring^5^, consistent with developmental programming in male germ cells that extends well into early post-natal life^6^. Therefore, nutritional modulation during early life affects testicular development, with apparent epigenetic effects on post-pubertal sperm function.

We reported increased cholesterol/steroid biosynthesis and Sertoli cell maturation in testicular tissues of high-diet bulls at 16 and 24 wk, respectively^7^. Although a wide range in pre-pubertal diets had no significant effects on routine analyses of sperm morphology and function^8^, molecular-level analyses should be done, to ensure no deleterious effects are transmitted to the next generation. Our objective was to determine effects of pre-pubertal dietary modulations on post-pubertal testes of Holstein bulls.

## Results

### ***mRNA*** profile of testes

Approximately 340 × 10^6^ reads were obtained from the 24 libraries sequenced. On average, 14,357,890 (SD = 2,267,076) reads were obtained per library and 13,565,597 (SD = 2,148,261) mapped to the Ensemble gene annotation database. On average 13,710,936 (SD = 3,098,630), 13,120,341 (SD = 1,219,498) and 13,865,513 (SD = 1,929,109) reads were mapped from HD, MD and LD groups respectively. A total of 18,288 genes were detected in testicular tissue and their Ensembl IDs are provided (Supplementary Dataset 1). On average, the most abundant gene in the testicular transcriptome was cytochrome c oxidase subunit I (*COX1*), a mitochondrial gene (ENSBTAG00000043561), representing ~2.9% of total reads. Other abundant genes included cytochrome c oxidase subunit 3 (*COX3*, ENSBTAG00000043560), ATP synthase F0 subunit 6 (*ATP6*, ENSBTAG00000043584) and cytochrome b (*CYB*, ENSBTAG00000043550), all mitochondrial genes.

### Differentially expressed genes

**Figure 1:**
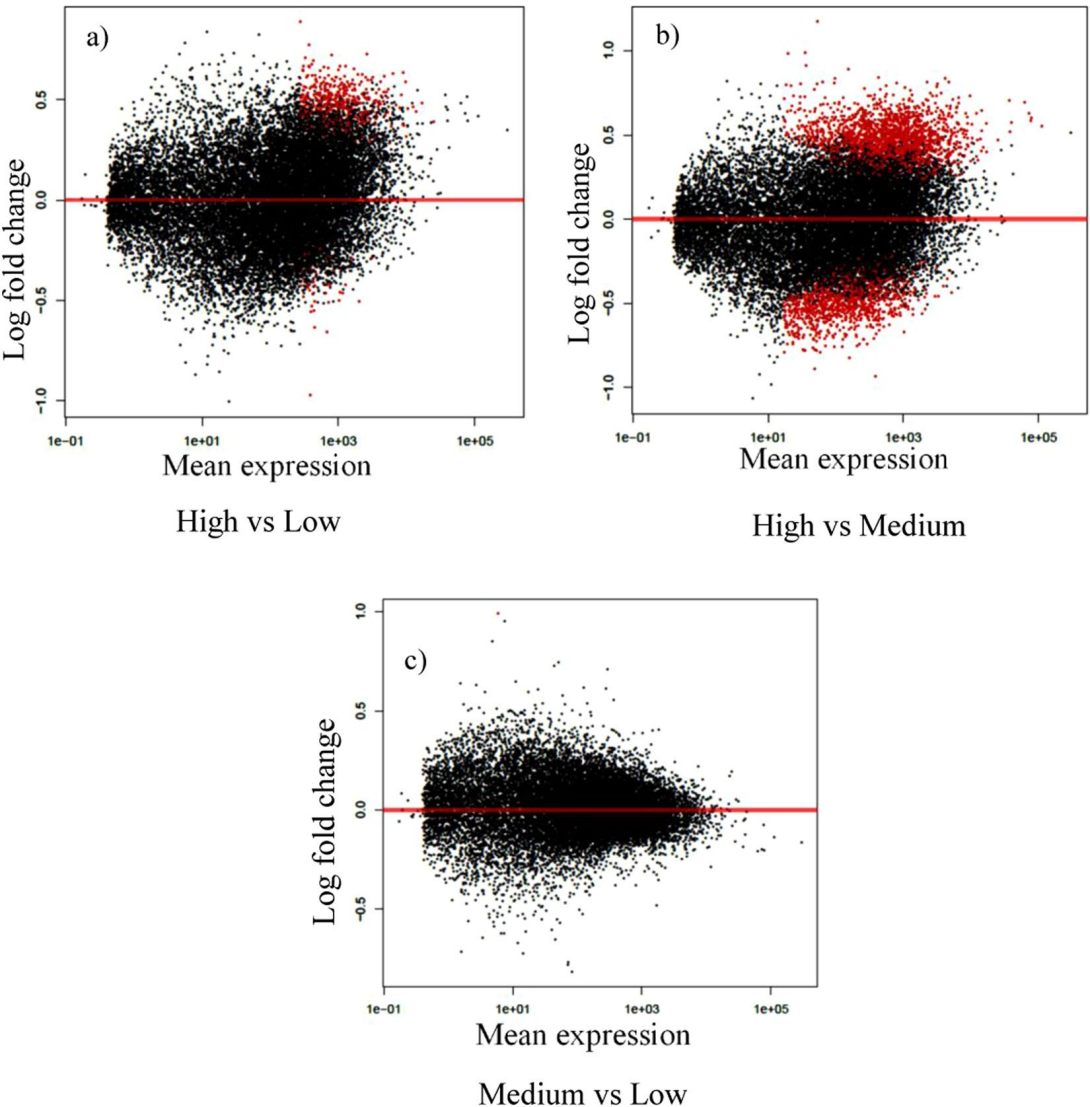
MA plots (visual interpretation of genomic data) for genes in testicular tissue of bulls fed various diets: (**a**) high vs low, (**b**) high vs medium and (**c**) medium vs low. Differentially expressed genes (P < 0.1) are highlighted in red. X-axis: mean expression, Y-axis: log fold change.

In general, of 18288 genes expressed, 2960 were differentially expressed (P < 0.1) between HD and MD groups compared to 496 in HD versus LD groups (Supplementary Datasets 2 and 3). A scatterplot denoting transcriptomic differences across diet groups is provided (Supplementary File 1). To visualise extent of differential expression and relationship between mean gene expression levels and log2fold changes, MA plots were generated (Fig. 1). Of the 496 DEGs in the HD versus LD analysis, 446 genes were upregulated in the HD group (genes with higher log2fold change were: *COX5B, COX6B1, ATP5J, ATP5G1, MRPL11* and *HSD17B1)*. When comparing HD and MD groups, of the 2960 DEGs, 1828 genes were upregulated in the HD group (genes with higher log2fold change: *COX2, COX3, COX5B, HSD17* and *CCNA1*). The log2fold changes varied from ± 0.21 to ± 1.17 among all DEGs. No genes were differentially expressed between MD and LD groups. Therefore, all further analyses were done using two datasets, HD vs LD and HD vs MD groups.

### Gene Ontology term enrichment

To systematically examine enriched genes associated with differential pre-pubertal diets on post-pubertal testes, gene ontology (GO) analysis was done using the functional annotation and clustering option in DAVID. “Mitochondrial translation initiation” and “elongation” were the major biological functions enriched in HD vs LD and HD vs MD groups. In the HD vs MD group comparison, “mitotic nuclear division”, “cytoplasmic translation” and “ATP synthesis coupled protein transport” were also enriched. Complete lists of GO terms enriched in HD vs LD and HD vs MD datasets are provided in Tables 1 and 2, respectively.

**Table 1:** GO terms enriched (P < 0.05) using DAVID, in testicular tissue of bulls fed high- vs low-nutrition diets.

GO term	Count	P value
Mitochondrial translational initiation	22	9.81E-12
Mitochondrial translational elongation	22	9.81E-12
Translation	20	2.459E-04
Mitochondrial translation	10	0.001
ER to Golgi vesicle-mediated transport	10	0.021

**Table 2:** GO terms enriched (P < 0.05) using DAVID in testicular tissue of bulls fed high- vs medium-nutrition diets.

GO term	Count	P value
Translation	99	9.36E-30
Mitochondrial translational elongation	45	8.63E-11
Mitochondrial translational initiation	44	3.36E-10
Mitochondrial translation	25	5.12E-05
Cytoplasmic translation	16	9.04E-04
Mitotic nuclear division	38	0.00
ATP synthesis coupled proton transport	14	0.00
Hydrogen ion transmembrane transport	17	0.00

### Over-represented KEGG pathways

Eight pathways were associated with HD vs MD groups and six pathways in HD vs LD groups when considering genes (P < 0.1, Tables 3 and 4). “Oxidative phosphorylation” and “ribosome pathways” were major pathways enriched in both datasets.

**Table 3:** KEGG Pathways enriched in genes differentially expressed (P < 0.05) in testicular tissue of bulls fed high- vs medium-nutrition diets.

KEGG term	Count	P value
Ribosome	99	1.20E-44
Oxidative phosphorylation	77	2.03E-22
Parkinson’s disease	77	3.81E-20
Alzheimer’s disease	75	3.10E-14
Huntington’s disease	80	7.77E-15
Non-alcoholic fatty liver disease (NAFLD)	58	9.68E-08
Proteasome	24	1.56E-05
Protein processing in endoplasmic reticulum	45	0.04

**Table 4:** KEGG Pathways enriched in genes differentially expressed (P < 0.05) in testicular tissue of bulls fed high- vs low-nutrition diets.

KEGG term	Count	P value
Oxidative phosphorylation	26	3.08E-11
Ribosome	25	5.87E-11
Parkinson’s disease	26	7.51E-11
Alzheimer’s disease	27	1.66E-10
Huntington’s disease	27	8.22E-10
Non-alcoholic fatty liver disease (NAFLD)	19	1.68E-05

In addition to above, oxidative phosphorylation and ribosome pathways were enriched in the upregulated genes from both datasets (HD vs MD and HD vs LD, Supplementary Files 2 and 3). However, nine downregulated pathways were identified in the HD vs MD dataset (Supplementary File 4). Major pathways identified were focal adhesion, regulation of actin cytoskeleton and axon guidance.

### PCR validation of differentially expressed genes

**Figure 2:**
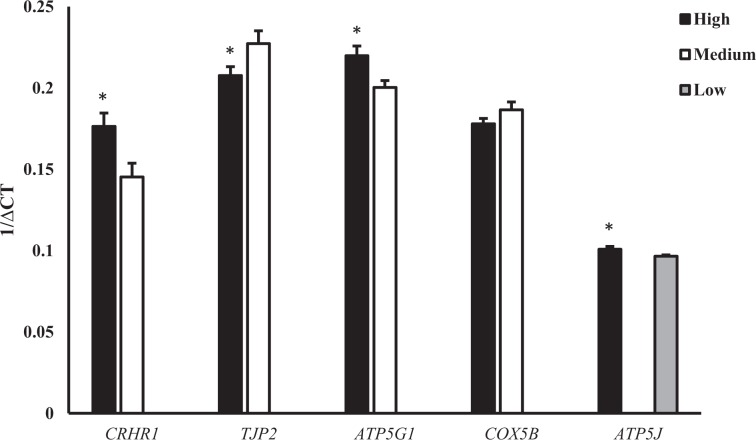
qPCR validation of differentially expressed genes in testes of bulls in high vs medium and high vs low datasets. Data are mean ± SEM. X-axis: official gene ID, Y-axis: 1/∆CT.

Five genes were selected from the list of differentially expressed mRNA for validation (Table 5). We were able to successfully validate four (*CRHR1, TJP2, ATP5G1* and *ATP5J*) of the five genes selected (P < 0.1, Fig. 2).

**Table 5:** Primer sequences used for validation of differentially expressed genes in testicular tissue of Holstein bulls fed various diets.

Gene	Abbreviation	Primer	Product size	Accession No.
Glyceraldehyde-3-phosphate dehydrogenase	GAPDH	F: GCATCGTGGAGGGACTTATGA	67	NM_001034034.2
		R: GGGCCATCCACAGTCTTCTG		
ATP synthase, H + transporting, mitochondrial F0 complex, subunit F	ATP5J	F: GCCCCAGTTCGCTTTGTTTT	102	NM_174717.3
		R: CCGAGTCAGCGTCCTATTCC		
Tight junction protein 2	TJP2	F: CAAAACCAGCCCAGAGAGACA	103	NM_001102482.1
		R: TCCTCCCGACTCTTCCGTAG		
Corticotropin releasing hormone receptor 1	CRHR1	F: TGAGAAGTGCTGGTTTGGCA	123	NM_174287.1
		R: GTCATGAGGATGCGGACGAT		
Cytochrome c oxidase subunit 5b	COX5B	F: ATGGCGTCAAGGTTACTCCG	77	NM_001034046.2
		R: GAGACTCCATTTGGACCCCG		
ATP synthase membrane subunit c locus 1	ATP5G1	F: GAGTCAGTCACCTTGAGCCG	142	NM_176649.3
		R: CCCCAGCCGAGAAGAACATT

### Sperm progressive motility and mitochondrial membrane potential (MMP)

**Figure 3:**
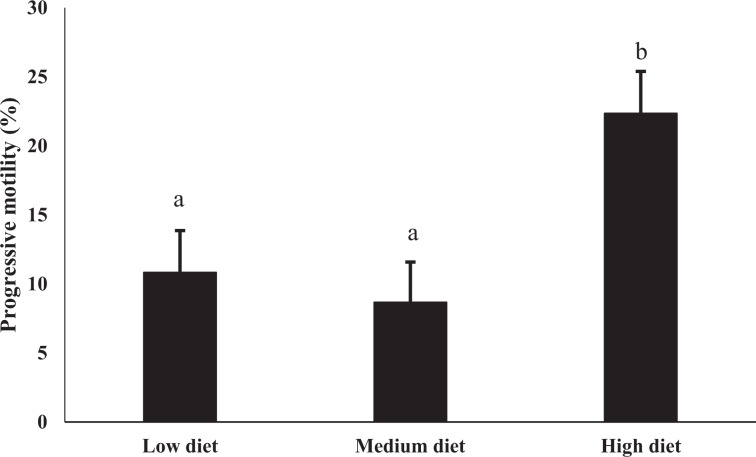
Progressive motility of frozen-thawed sperm collected from post-pubertal Holstein bulls at two time points (55–59 and 69–71 wk). ^a,b^Means without a common superscript differed (P < 0.1).

**Figure 4:**
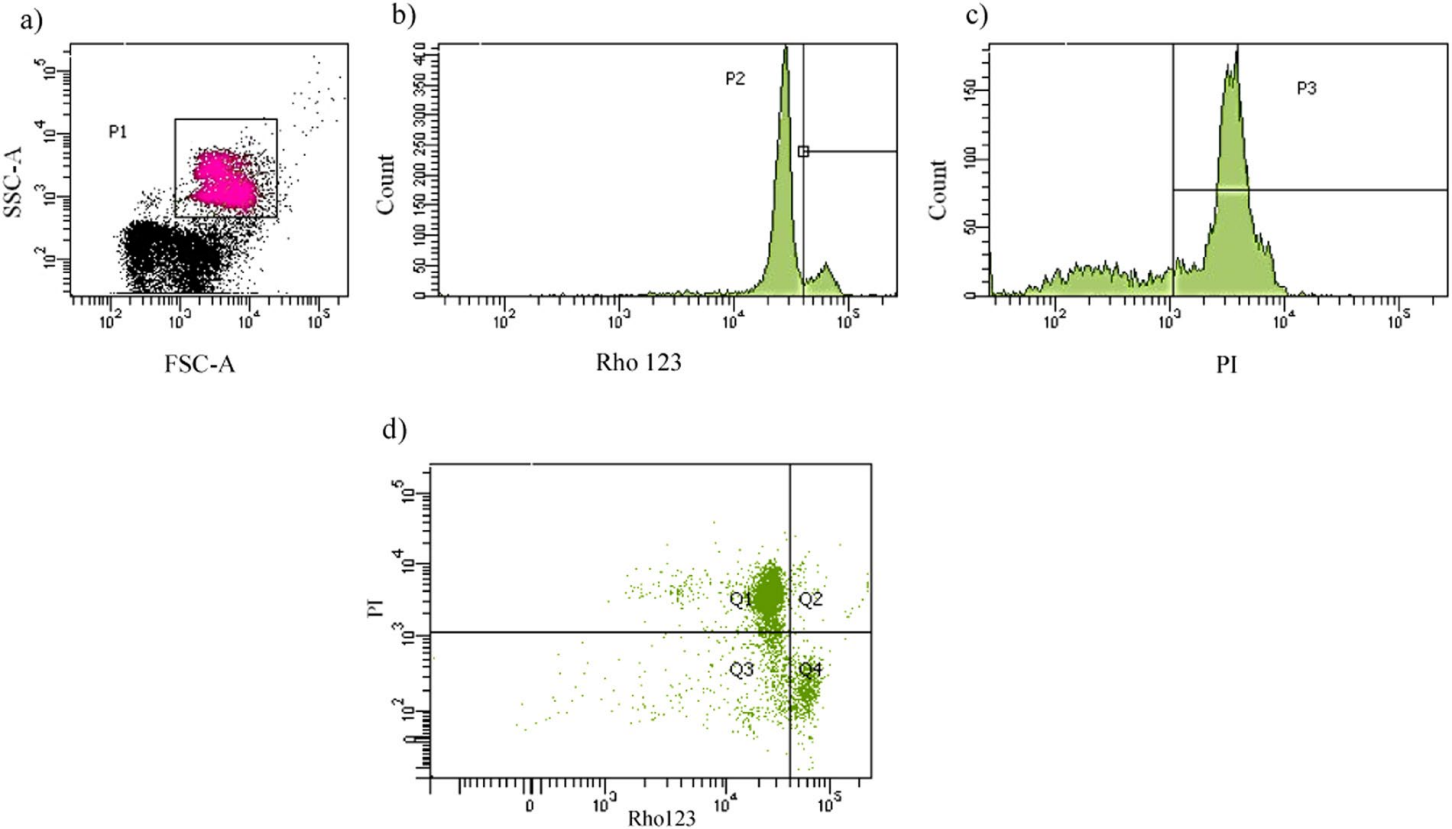
Representative images of flow cytometry using sperm stained with Rhodamine 124 and PI to evaluate mitochondrial membrane potential (MMP). (**a**) Represents the gating to establish the population (P1); (**b**) histogram for SYBR14; (**c**) histogram for PI; (**d**) shows the four population of cells- Lower right quadrant (Q4): Rh123 + /PI−, live sperm with normal mitochondrial function, Lower left quadrant (Q3): Rh123−/PI−, live sperm losing mitochondrial function, Upper left quadrant (Q1): Rh123−/PI+, dead/necrotic sperm.

Sperm of HD bulls had greater (P < 0.1) post-thaw progressive motility than sperm from MD or LD bulls (22.3 ± 3.0, 8.7 ± 2.9 and 10.8 ± 3.0 respectively, Fig. 3). There were also differences across dietary treatments in percentages of Rhl23-/PI- (P < 0.1) and Rhl23-/PI + sperm (P < 0.05; Table 6). Percentage of Rhl23−/PI− sperm (live sperm loosing mitochondrial function) was higher in MD vs HD groups (15.1 ± 2.6 vs 8.6 ± 0.9). However, there was no significant difference among groups for percentage of Rhl23 + /PI- sperm (live sperm with normal mitochondrial function). The percentage of Rhl23-/PI + sperm (dead or necrotic sperm) in the MD was 66.2 ± 3.5 and was lower (P < 0.05) than both LD and HD groups (78.6 ± 2.2 and 78.7 ± 2.2, respectively). A representative image of flow cytometry explaining sperm populations is provided (Fig. 4).

**Table 6:** Comparison of flow cytometry results of sperm from bulls fed (low-, medium- or high-nutrition diets). Data are mean ± SEM.

Diet	Low	Medium	High
Rhl23 + /PI−	6.9 ± 1.2^a^	14.2 ± 3.2^a^	11.2 ± 2.8^a^
Rhl23−/PI−	13.3 ± 1.6^ab^	15.1 ± 2.6^a^	8.6 ± 0.9^b^
Rhl23−/PI+	78.6 ± 2.2^a^	66.2 ± 3.5^b^	78.7 ± 2.2^a^

^a,b^Within a row, means without a common superscript differed (P < 0.05).

## Discussion

To provide a more comprehensive assessment of post-pubertal effects of pre-pubertal feeding, RNA-sequencing was done on testicular tissues collected at 72 wk of age. To our understanding, this is the first study to evaluate effects of differential feeding during early pre-pubertal period on the post-pubertal bovine transcriptome using a next-generation sequencing technique. Bulls on the HD (fed supplemental protein and energy) had greater oxidative phosphorylation and mitochondrial protein synthesis within their testes compared to LD and MD bulls. Furthermore, sperm from HD bulls had greater progressive motility and a greater percentage had normal mitochondrial function compared to sperm from MD bulls.

Oxidative phosphorylation (OXYPHOS) occurs in mitochondria, generating nearly 80% of cellular energy. OXYPHOS, the functional unit of mitochondria, connects the electron transport chain (ETC) to cell respiration and ATP synthesis. Enzyme complexes constituting OXYPHOS are comprised of mitochondrial and nuclear encoded polypeptides^9^. Elevated mitochondrial and cytoplasmic translation in HD vs MD were indicative of increased protein availability for OXYPHOS formation. A similar study in Holstein bulls also identified increased OXYPHOS in adipose tissue along with enhanced testes development, cholesterol and androgen biosynthesis in the testes of pre-pubertal bulls fed high vs low planes of nutrition^10–12^.

The integral part of OXYPHOS, the ETC, is composed of five multi-protein complexes and two electron carriers (ubiquinone and cytochrome) transporting electrons to oxygen, creating a proton motive force. The resulting membrane potential enables energy production when these protons flow back via ATP synthase^13^. Proton transfer at sites other than ATP synthase reduces mitochondrial efficiency, but is critical for ROS production. Testicular mitochondria consume less oxygen than other tissues to maintain the same electric potential and have higher phosphorylation efficiency with age^14^. In mature testes, spermatocytes and spermatids use OXYPHOS, due to limited glucose availability in seminiferous tubule fluid, whereas Sertoli cells, spermatocytes and mature sperm have high glycolytic activity^15,16^. The majority of upregulated genes in HD (vs MD and LD) were significantly enriched for OXYPHOS.

In sperm, mitochondrial activity has been associated with sperm motility^17^, viability^18^, capacitation^19^ and fertilization potential^20^. Within germ cells, mitochondrial ATP synthesis was reported to regulate the apoptotic pathway^21^. Higher MMP has been associated with increased sperm motility and production, as sperm require ATP^17^. In addition, MMP is correlated with activity of the ETC and ATP synthase (components of the OXYPHOS). Increased mitochondrial activity beyond its threshold could induce oxidative stress, which could alter DNA. The majority of genes involved in the ETC had high expression in HD bulls compared to MD and LD bulls. In addition, major mitochondrial functions, including ATP synthesis coupled protein transport, hydrogen ion transmembrane transport, mitochondrial electron transport and oxidation reduction processes were altered in HD vs MD bulls.

A positive association between mitochondrial function and motility has been reported in bovine sperm^22^. In our study, sperm from HD bulls had greater progressive motility (PM) compared to sperm from MD and LD bulls; the association between motility and mitochondrial function, reflected our gene expression study. Furthermore, positive associations between OXYPHOS, ETC and sperm motility have been reported^18,23,24^. We also evaluated MMP, for which Rhodamin123, a mitochondria-specific fluorescent dye in combination with PI, was used^25^. When comparing live cells, a higher percentage of sperm from MD bulls had lowered MMP than sperm from HD bulls. Surprisingly, percentages of dead/necrotic sperm were significantly elevated in both HD and LD groups compared to the MD group (Table 6). An increased percentage of dead sperm in our HD group was reported in our phenotypic study^8^. A possible explanation could be the increased ROS production favoured by the elevated mitochondrial function in high diet bulls promoting cell death^26^. Both progressive motility and MMP results were consistent with our gene expression study, where the majority of genes involved in mitochondrial function were upregulated in HD versus MD groups. Positive associations between MMP and motility have also been reported in sperm^22,27^.

In addition to altered ETC function, majority of genes associated with ribosome pathway were upregulated in HD vs MD groups. We reported that supplemental energy and protein in diets of pre-pubertal bull calves hastened puberty and increased both testes size and sperm production potential, with no significant differences in classical assessments of sperm quality or function^4,8^. The role of mitochondrial function in regulating various aspects of sperm function, including meiosis, spermatogenesis, energy for survival, motility, capacitation, maturation and quality control has been reviewed^28^. Increased mitochondrial activity is essential to support elevated sperm production potential previously reported in HD bulls. High ribosomal biogenesis is also intimately associated with cellular activities, including cell growth and division^29^. All genes involved in the ribosome pathway were upregulated in the HD group, implying high ribosomal biogenesis.

In addition to its specific role in sperm function, mitochondria also have an endocrine role in testes^30^. The rate limiting and first step in steroid biosynthesis, converting cholesterol to pregnanolone, takes place within mitochondria and is catalysed by the enzyme cytochrome P450 side cleavage^31^. StAR promotes cholesterol movement in mitochondria (from outer to inner membranes) and after pregnanolone is formed, it is transported to endoplasmic reticulum to complete steroid biosynthesis^32^. Increased steroid production (mainly testosterone) is critical for male reproductive development and sperm production.

In addition to being critical for cell growth and metabolism, mitochondrial function is closely associated with epigenetics^33,34^. Epigenetic changes can be induced by various factors in the environment, e.g. nutrition, that can alter gene expression by incorporating changes into the epigenome. The epigenome is a record of chemical changes on the DNA and histone proteins that may be transferred to the next generation^35,36^. Substantial epigenetic changes, including methylation of DNA methylation and modification of histones, use various metabolic intermediates, including s-adenosyl methionine (SAM) and acetyl coA^34^. Based on the energy status of a cell, these metabolites can vary and are to an extent regulated by mitochondrial function. For instance, energy production within mitochondria converts dietary calories into ATP, acetyl coA, SAM and reduced NAD. With a high-energy diet, there is phosphorylation and acetylation of chromatin by ATP and acetyl-CoA, thereby making nDNA more available for transcription and replication. However, if dietary energy is limited, phosphorylation and acetylation of chromatin do not proceed, thereby supressing gene expression. Methylation of DNA facilitated by SAM can also be regulated by mitochondrial function^37,38^. Thus, alterations in mitochondrial function could cause epigenetic changes with long-term consequences. In addition, recent studies in our lab are indicative of DNA methylation changes in sperm of bulls fed differential pre-pubertal diets (unpublished data). It is possible that the sperm epigenome keeps memory of diet during pre-pubertal period in genes critical for sperm function. However, more and more studies in humans have demonstrated the ability of paternal environment to influence the epigenetic/non-genetic information transmission carried by sperm cells and modify the trajectory of offspring^39–41^.

To summarise, enhanced pre-pubertal feeding in Holstein bulls upregulated genes associated with mitochondrial function, including oxidative phosphorylation, ETC and mitochondrial protein synthesis. In addition to enhanced mitochondrial function, elevated mitotic activity and ribosome synthesis could have supported greater sperm production potential of HD bulls, as evidenced in our phenotypic study. In addition to greater progressive motility, percentage of live sperm losing mitochondrial function was much lower in HD vs MD groups. We concluded that enhanced pre-pubertal nutrition followed by a control diet enhanced mitochondrial function and sperm quality in Holstein bulls.

## Materials and Methods

### Ethics approval

This experiment was conducted in accordance with the guidelines of the Canadian Council on Animal Care and was reviewed and approved by the Lethbridge Research Centre Institutional Animal Care Committee.

### Animals and treatments

Tissues were derived from bulls on three dietary treatments, which have been described in detail^4^. Briefly, 1-wk-old Holstein bull calves (n = 24) were randomly allocated to 3 groups that were fed high-, medium- or low-diets (HD, MD and LD, respectively) from 2 to 32 wk of age. From 2–8 wk of age, HD, MD and LD calves were fed milk (8, 6 or 4 L/d, respectively), followed by transition to a barley silage-based diet. The HD (n = 8 calves) consisted of 49.7% rolled barley, 9.7% rolled corn, 7.6% canola meal, and 7.6% soybean meal (20.0% crude protein [CP] and 67.9% total digestible nutrients [TDN]). The MD (n = 8 calves) was the control and contained 4.8% rolled barley, 4.8% rolled corn, 3.8% canola meal, and 3.8% soybean meal (17.0% CP and 66.0% TDN). The LD (n = 8 calves) was barley silage (plus premix, but no concentrate), with 12.2% CP and 62.9% TDN. All diets contained 1.6% vitamin-mineral premix (as fed), with CP and TDN reported on a dry matter basis. The HD was fed *ad libitum* and based on voluntary intake; the same amount of feed (on an as-fed basis) was offered to MD and LD calves. Calves were on their respective diets until 31 wk of age, after which they were all fed the MD. At 72 wk, bulls were slaughtered, and testicular tissue collected and preserved. Testicular parenchyma was cut into cubes (~10 mm in each dimension) and snap-frozen on dry ice. This experiment was conducted in accordance with Canadian Council on Animal Care guidelines and was reviewed and approved by the Lethbridge Research Centre institutional animal care committee.

### Bull phenotype

Body weights, scrotal circumference and paired testes volume from 11–71 wk of age have been reported^4^. Briefly, high-diet bulls had greater body and paired testes weights at slaughter (71 wk). Sperm production potential was significantly greater in HD versus LD bulls, but there were no significant differences among groups regarding age when post-thaw semen first achieved minimum standards (based on progressive motility and morphology) or on sperm viability, protein content or *in vitro* fertilization potential^8^.

### Total ***RNA*** extraction

Testicular tissue (75 mg) was homogenized and total RNA extracted using Trizol reagent, as per manufacturer’s instructions (Invitrogen, Canada, Burlington, ON, Canada). Total RNA was suspended in water, quality and quantity measured using a Nanodrop via spectrophotometry (2000/2000c, Thermo Fisher Scientific, Waltham, MA, USA). The RNA integrity number (RIN) was assessed on an Agilent 2100 bioanalyzer using an Agilent RNA 6000 Nano kit (Agilent Technologies, Santa Clara, CA, USA).

### ***mRNA*** sequencing of testicular tissue

#### RNA-sequencing library preparation and sequencing

For each sample (n = 24), total RNA (1 µg) was used to create mRNA libraries using the TruSeq Stranded mRNA Library Prep Kit (Illumina Inc., San Diego, CA, USA) and unique adapter indices (Illumina) at the University of Lethbridge sequencing facility (Lethbridge, AB, Canada). Libraries were sequenced as 75 base pair single end reads using the NextSeq. 500 system (Illumina Inc.). For this purpose, individual libraries were pooled to obtain a single library by diluting individual libraries to a final concentration of 4 nM and adding equal volumes of all samples (n = 24). Base calling and demultiplexing was performed with CASAVA 1.9 (Illumina Inc.), using default settings. Quality of sequencing reads was assessed using FastQC software^42^. Quality of sequencing libraries was considered adequate to proceed with downstream analysis without adapter and quality trimming. Very limited adapter contamination was identified.

#### Mapping and annotation of reads

Sequencing reads were mapped to the bovine genome (*Bos taurus*, UMD3.1.87, downloaded from Illumina iGENOME website) using TopHat v2.0.10^43^ and with Bowtie v.1.1.2^44^ used as an internal aligner. Mapping rate was uniformly good, with >93% of reads successfully mapped to the genome.

#### Detection of differentially expressed genes

Counts of reads mapping to genes were obtained using featureCounts software from Subread package^45^. Count data were loaded into R, with normalization and variance stabilizing transformation^46^ applied using DESeq2 package. Normalized and variance stabilized data were used for quality control and sample clustering analyses. Euclidean sample distances were calculated in R and sample clustering was done using hclust^47^ function in R. Sample to sample distances were visualized as heatmaps. Principal components plots were made with PCAplot function in DESeq2 package^48^.

Genes differentially expressed between diets were detected using Wald tests on normalized count data. Experimental conditions were compared with one-way ANOVA; thereafter, results for specific contrasts (HD vs LD, HD vs MD and MD vs LD) were extracted. Correction procedures for multiple comparisons were done with Benjamini-Hochberg correction implemented in R. Genes with adjusted P-values < 0.1 were considered differentially expressed.

### Functional analysis

The Ensembl identities of DEGs from HD vs LD and HD vs MD analyses were uploaded to Database for Annotation, Visualization and Integrated Discovery (DAVID) to enrich for Gene Ontology (GO) Biological process and cellular components^49^. For most analyses, we used the functional annotation and clustering option and reported all GO terms that were P < 0.05 and molecule number ≥2.

### Quantitative real-time polymerase chase reaction (RT-PCR)

RT-PCR was performed as described previously^7^. In brief, reverse transcription and subsequently PCR with SYBR Green (Fast SYBR® Green Master Mix; Applied Biosystems, Foster City, CA, USA) was done to validate mRNA expression of five differentially expressed genes: TJP2, COX5B, ATP5J, ATP5G1 and CRHR1. The National Center for Biotechnology and Information (NCBI) was used to design PCR primer sequences (Table 5), with primers purchased from Thermo Fisher Scientific (Waltham, MA, USA). RNase-free DNase I (Thermo Fisher Scientific) was added to total RNA (1 µg). After incubation at RT for 15 min at RT, 25 mM EDTA was added and the mixture hated to 65 °C for 10 min to cause denaturation. Then, High Capacity cDNA Reverse Transcription Kit and random hexamers (Applied Biosystems) were used for reverse transcription. For all primers, cDNA was diluted 1:10 and was efficiently used for PCR. All amplification reactions were done in 96 well plates (Applied Biosystems), as follows: 95 °C for 10 min, followed by 40 cycles of 95 °C for 15 s and 59 °C for 1 min and finally dissociation (55–95 °C). Every reaction well contained: Fast SYBR green Master mix- 7.5 μl, forward primer (20 pmol/μl)- 0.5 μl, reverse primer (20 pmol/μl)- 0.5 μl, cDNA template- 0.5 μl and water-1 μl. All reactions were done in duplicate, with inclusion of appropriate controls (non-RT and no-cDNA). Purity was confirmed by melt curve analysis. Glyceraldehyde-3-phosphate dehydrogenase (GAPDH;^50^) was used as a housekeeping gene to normalize Cq (mean threshold cycle) and (∆) Cq values were expressed as 1/ ∆Cq for ease of understanding interpretation of gene expression.

### Evaluation of sperm progressive motility

Sperm collected from a subset of three bulls/treatment at two time points (55–59 and 69–71 wk) were analyzed; this selection of bulls and time points was based on availability of cryopreserved semen samples. Sperm progressive motility was evaluated using computer assisted sperm analysis (CASA), as described^51^. In brief, a small aliquot of semen (4 μL) was loaded into a prewarmed chamber slide (20 μm deep; Leja Products, Nieuw-109, Vennep, the Netherlands) and immediately analyzed under 400X magnification for progressive motility. Seven microscopic fields per sample were analyzed and the frame rate was 60/s.

### Evaluation of sperm mitochondrial membrane potential (MMP)

Sperm samples used for motility analysis were also assessed for mitochondrial membrane potential with a combination of Rhodamine 123 and Propidium Iodide (PI). Rh123/PI dual fluorescent staining and flow cytometry were done as described^25^. In brief, frozen-thawed semen was washed twice in PBS (700 x G for 5 min) and sperm concentration adjusted to 5 × 10^6^/mL. Then, Rh123 (Molecular Probes, Eugene, OR, USA) was added to the sperm suspension at a final concentration of 5 µg/mL and incubated for 5 min at 37 °C in the dark. To remove non-specific binding, sperm were washed in PBS (700 x G for 10 min), followed by addition of propidium iodide (PI, Invitrogen, Canada, Burlington, ON, Canada) at a final concentration of 5 µg/mL and incubated for 5 min in the dark. The PI staining was performed for concurrent evaluation of both viability and mitochondrial function. Flow cytometry analysis (FACScan, Beckon Dickinson, San Jose, CA, USA) was done with an argon excitation laser (488 nm) and >10 × 10^3^ events recorded for each sample.

### Statistical analyses

Data for RT-qPCR, motility and mitochondrial membrane potential were analysed using R software (v 3.4.3;^47^). Depending on number of groups involved, ANOVA or independent Student’s *t*-tests were used to detect differences, with P < 0.1 defined as significant.

## Supplementary information

Supplementary information .

Dataset 1.

Dataset 2.

Dataset 3.

## Data Availability

All data generated or analysed during this study were deposited in the publicly available NCBIs Gene Expression Omnibus Database GSE137690 https://www.ncbi.nlm.nih.gov/geo/query/acc.cgi?acc = GSE137690.
