# Supplementary material for: Enhanced pre-pubertal nutrition upregulates mitochondrial function in testes and sperm of post-pubertal Holstein bulls
Source: Sci Rep. 2020 Feb 10;10:2235. doi: 10.1038/s41598-020-59067-3 (PMC7010748; doi:10.1038/s41598-020-59067-3)
Supplement: Supplementary file 1 — Supplementary information . [file 41598_2020_59067_MOESM1_ESM.docx]

**Enhanced pre-pubertal nutrition upregulates mitochondrial function in the testes and sperm of post-pubertal Holstein bulls**

Chinju Johnson^1^, Alysha Dance^1^, Igor Kovalchuk^2^, John Kastelic^1^ and Jacob Thundathil^1*^

^1^Department of Production Animal Health, Faculty of Veterinary Medicine,

University of Calgary, Calgary, AB T2N 4N1, Canada

^2^Department of Biological Sciences, University of Lethbridge,

Lethbridge, AB, TIK 3M4, Canada

1. Supplementary file 1: Scatterplot with transcriptomic differences across bulls from high, medium and low-diet groups using principal component analysis (PCA) (Axes: PC1 28%, PC2 15%).


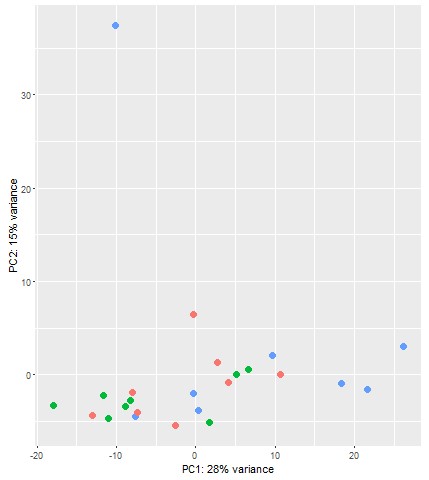

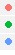


**Group**

**Low**

**Medium**

**High**

1. Supplementary file 2: KEGG Pathways enriched in the upregulated DEGs between High vs medium diet groups at p <0.05.

| **KEGG Term** | **Count** | **P value** |
| --- | --- | --- |
| Ribosome | 99 | 3.10E-62 |
| Oxidative phosphorylation | 76 | 7.86E-34 |
| Parkinson's disease | 73 | 1.66E-28 |
| Huntington's disease | 73 | 3.96E-21 |
| Alzheimer's disease | 68 | 7.78E-20 |
| Non-alcoholic fatty liver disease (NAFLD) | 51 | 6.14E-11 |
| Proteasome | 24 | 4.47E-09 |
| Metabolic pathways | 161 | 7.65E-06 |
| Cardiac muscle contraction | 19 | 0.04 |

1. Supplementary file 3: KEGG Pathways enriched in the upregulated DEGs between High vs low diet groups at p <0.05.

| **KEGG term** | **Count** | **P value** |
| --- | --- | --- |
| Oxidative phosphorylation | 26 | 9.66E-13 |
| Parkinson's disease | 26 | 2.43E-12 |
| Ribosome | 25 | 2.15E-12 |
| Alzheimer's disease | 27 | 5.00E-122 |
| Huntington's disease | 27 | 2.61E-11 |
| Non-alcoholic fatty liver disease (NAFLD) | 19 | 1.70E-06 |

1. Supplementary file 4: KEGG Pathways enriched in the downregulated DEGs between High vs medium diet groups at p <0.05.

| **KEGG term** | **Count** | **P value** |
| --- | --- | --- |
| Pathways in cancer | 46 | 2.54E-04 |
| Focal adhesion | 29 | 5.78E-04 |
| Proteoglycans in cancer | 26 | 1.05E-02 |
| Regulation of actin cytoskeleton | 26 | 1.05E-02 |
| Axon guidance | 18 | 0.03 |
| Signaling pathways regulating pluripotency of stem cells | 19 | 0.02 |
| Platelet activation | 18 | 0.03 |
| Small cell lung cancer | 14 | 0.03 |
| Rap1 signaling pathway | 23 | 0.04 |
